# A Dire Presentation of Diabetic Ketoacidosis with "Black Esophagus

**DOI:** 10.7759/cureus.4761

**Published:** 2019-05-27

**Authors:** Aun R Shah, Marc Landsman, Nisheet Waghray

**Affiliations:** 1 Internal Medicine, Case Western Reserve University School of Medicine / Metrohealth Medical Center, Cleveland, USA; 2 Gastroenterology & Hepatology, Case Western Reserve University School of Medicine / Metrohealth Medical Center, Cleveland, USA

**Keywords:** black esophagus, diabetic ketoacidosis, necrotizing esophagitis, acute esophageal necrosis, candida, aen, diabetes, dka

## Abstract

Acute esophageal necrosis (AEN) is a rare life-threatening illness that is being increasingly recognized in the past two decades. It usually develops in the setting of severe systemic illness due to a combination of tissue hypoperfusion, impaired mucosal defenses and gastric reflux. The most common presentation is with upper gastrointestinal bleeding complicating diabetic ketoacidosis, sepsis, pancreatitis, trauma, shock, renal failure, alcohol poisoning or other states of hemodynamic compromise. The classic finding on endoscopy is of necrosis of the distal esophagus with a sharp transition to normal gastric mucosa at the gastroesophageal junction. Management is aimed at treating the underlying insult and providing supportive care. We report a case of "black esophagus" complicating an episode of diabetic ketoacidosis in a 34-year-old male. The patient was treated with broad-spectrum antibiotics, antifungals and a high-dose proton pump inhibitor in addition to the treatment of ketoacidosis. No serious acute or long-term complication was identified and follow-up endoscopy showed resolution of necrosis.

## Introduction

Acute esophageal necrosis (AEN), also known as "black esophagus" or necrotizing esophagitis, is a rare entity which has been described as far back as the 1960s, but it wasn’t until the 1990s that it gained widespread recognition [1-2]. Classically described as black necrosis of the distal esophagus with sharp demarcation at the gastro-esophageal junction (GEJ), necrotizing esophagitis has however been known to affect the proximal and middle third of the esophagus [3]. It is a disorder of complex pathophysiology with multifactorial etiology, leading to a common final pathway of impaired mucosal barriers, ischemic injury and reflux of gastric content, resulting in severe esophageal injury and necrosis. Hypertension, diabetes mellitus, malignancy, male sex, older age, chronic kidney disease, alcohol abuse and cardiovascular disease are associated with risk of developing AEN [3-4]. Treatment is mostly supportive and is aimed at minimizing esophageal injury whilst addressing instigating insults [2]. The most serious acute complication is perforation of the esophagus which carries a high risk of morbidity and mortality [5-6]. The most common serious long-term sequela of AEN is stricture formation which can complicate up to 10% of all cases [2]. Even with adequate management prognosis is poor and a mortality rate as high as 36% has been reported in literature [7-8].

We present a case of "black esophagus" and candida esophagitis complicating diabetic ketoacidosis (DKA).

## Case presentation

A 34-year-old male with poorly controlled diabetes mellitus (hemoglobin A1c>9.1%) was admitted to the medical intensive care unit with diabetic ketoacidosis, sepsis and melena. The patient had an extensive past medical history which included multiple admissions for diabetic neuropathy, gastroparesis, neurogenic bladder, pyelonephritis, chronic diarrhea due to pancreatic insufficiency, lower extremity amputations for gangrene and methicillin-resistant* Staphylococcus aureus *(MRSA) osteomyelitis. Also, of note was an admission four months prior to this encounter for emphysematous gastritis and esophageal candidiasis.

The patient reported having malaise for one week with congestion, chills and dry cough. His symptoms progressively worsened and he started having fevers with rigors. Subsequently, the patient developed anorexia and stopped taking insulin. Two days prior to admission he also began having multiple episodes of melena each day but had no abdominal distension, hematochezia, vomiting or hematemesis. There was no dyspnea, loss of consciousness, limb weakness or chest pain. The patient presented to the emergency room after he had a syncopal episode and fell at home.

On admission his blood pressure was 84/48 mmHg, heart rate was 100 beats per minute, his temperature was 34.9 °C and oxygen saturation was 100% on room air. The examination was only remarkable for non-specific abdominal tenderness and a clean, well-healed stump from recent amputation. the pH was 6.926 with an anion gap of 19 and beta-hydroxybutyrate levels of 0.7 mmol/L. Treatment of diabetic ketoacidosis was initiated in the emergency room with normal saline infusion and intravenous insulin infusion. The patient was also found to have a white cell count of 28,800/uL with left shift and was started on vancomycin, meropenem with the addition of micafungin given the recent candida infection. Creatinine was elevated from a baseline of 1.7 mg/dL to 1 2mg/dL and fractional excretion of sodium was 1.5%. The patient received one unit packed red-blood-cell transfusion and planned to have upper gastrointestinal (GI) endoscopy as hemoglobin was 6g/dL with positive fecal occult blood testing. Coagulation studies were unremarkable. Rhinovirus PCR testing on nasopharyngeal swabs was positive. Nasal endoscopy performed by ear, nose and throat (ENT) surgeons showed evidence of viral illness but no signs of mucormycosis. Over the next few days, DKA was resolved but the patient continued to have unexplained fevers with negative blood cultures. He also required multiple blood transfusions to maintain acceptable hemoglobin levels. Serum *Helicobacter pylori *antibodies were negative.

Upper GI endoscopy was completed on the fourth day of admission, revealing a "black esophagus" starting at approximately 21 cm from incisors and extending proximally up to the GEJ. Tissue was noted to slough off easily but a normal mucosa could not be visualized. Squamocolumnar junction, diaphragmatic hiatus and GEJ were at 42 cm from the incisors. Additional findings included normal appearance of the bulb and second part of the duodenum as well as a normal gastric mucosa. Endoscopic biopsies of the black distal esophagus showed acute inflammatory exudates with necrotic cells and a nonspecific keratin stain pattern. Cytomegalovirus (CMV) and herpes tests were negative but numerous fungal hyphae were seen on methenamine silver staining.

Gastroenterology team recommended to continue with antimicrobial therapy and high dose proton pump inhibitors and also prescribed ongoing supportive care including good glycemic control, fluid resuscitation and enteral diet as tolerated. Hospital course was further complicated by *Staphylococcus hominis *bacteremia from the central venous line. However, overall clinical condition continued to improve and the patient was transferred to the general medical floor on the tenth day of admission. The patient was discharged to a skilled nursing facility on the thirteenth day of admission with plans to continue meropenem and micafungin for two more weeks. Follow up upper GI endoscopy showed resolution of necrosis, nevertheless, there was evidence of Los Angeles Grade D esophagitis. The patient had multiple subsequent admissions for chronic diarrhea, gastroparesis and fungal esophagitis.

## Discussion

"Black esophagus" or necrotizing esophagitis is a rare clinical entity formally recognized in the 1990s, that gained popularity after a series of cases were reported in 2007 [9-10]. It is a life-threatening condition characterized by acute necrosis of the distal esophagus sharply delineated with normal gastric mucosa at the GEJ. The etiology of AEN is poorly understood and likely multifactorial [11]. The hypothesized mechanism of injury is hypoperfusion and ischemia combined with an impaired mucosal barrier that leaves the mucosa defenseless against gastric reflux [12-13]. Tissue hypoperfusion explains the predilection for distal esophagus which is perhaps less vascularized than proximal or middle third; making it vulnerable to ischemic insults [14]. This hypothesis is also supported by the fact that AEN almost always develops in low perfusion states such as sepsis, congestive heart failure, renal failure, diabetic ketoacidosis, pancreatitis, hypothermia and shock [2]. Most of these conditions are also associated with vasculopathy, impaired blood supply and impaired mucosal barrier. Our patient presented with diabetic ketoacidosis, sepsis, shock and had a background of diabetic nephropathy as well as gastroparesis; thus, putting him at risk for tissue hypoperfusion, impaired mucosal defenses and reflux of gastric contents. Furthermore, he was found to be colonized by *Candida albicans*, which is frequently associated with AEN similar to other pathogens such as Klebsiella species, cytomegalovirus, herpes simplex virus [8, 15].

Comparable to our case, AEN most commonly presents with symptoms and signs of upper gastrointestinal bleeding in men who have multiple comorbidities [2]. Clinical presentation may also be with abdominal pain, emesis, dysphagia, nausea, fever, syncope or symptoms related to an underlying disorder and signs of systemic inflammatory response [8]. Endoscopy is diagnostic, showing findings as described previously, with distal to proximal involvement being the rule. Duodenal mucosal injury may also be present representing the shared blood supply of distal esophagus and the proximal duodenum [16-17]. Histology of involved esophagus illustrates severe inflammation and necrosis that may extend to the full thickness of esophageal wall [7]. Biopsy specimens should also be tested for fungal, bacterial and viral pathogens commonly associated with AEN.

Management of black esophagus is directed towards treating any underlying pathologies and providing supportive care. Treatment strategies include addressing hypoperfusion with intravenous fluid resuscitation, correction of anemia with blood product transfusion, adequate analgesia and initially avoiding enteral feeding. Parenteral feeding may be required until the oral diet can be advanced [6]. Aggressive acid suppression with high-dose proton pump inhibitors is recommended and can be continued beyond the resolution of acute necrosis to try and prevent long-term sequelae. Antibiotics, antifungals and antivirals may also be required as indicated. Empiric broad-spectrum antibiotics should be used with caution, however, as there are reports associating antibiotic use with the development of necrotizing esophagitis [18-19]. The most serious acute complication is perforation of the esophagus which have morbidity and mortality rates [5-6]. The most common long-term sequela of AEN is stricture formation which can complicate up to 10% of all cases [2]. Despite aggressive treatment, black esophagus carries a very poor prognosis. A mortality rate as high as 36% has been reported in the literature, however, this also depends on the underlying conditions [7-8]. 

## Conclusions

Diabetic ketoacidosis is a critical condition that can be further complicated by the development of necrotizing esophagitis. Prompt management of DKA and adequate supportive care can result in resolution of AEN and prove to be lifesaving. The case presented here emphasizes the importance of being mindful of necrotizing esophagitis as a cause of upper gastrointestinal tract bleeding in the context of DKA and corroborates various recommendations in the literature.
